# Analysis of Different Device Interactions in a Virtual Reality Task in Individuals With Duchenne Muscular Dystrophy—A Randomized Controlled Trial

**DOI:** 10.3389/fneur.2019.00024

**Published:** 2019-01-29

**Authors:** Bruna Leal de Freitas, Talita Dias da Silva, Tânia Brusque Crocetta, Thais Massetti, Luciano Vieira de Araújo, Shelly Coe, Helen Dawes, Fatima Aparecida Caromano, Carlos Bandeira de Mello Monteiro

**Affiliations:** ^1^Post-graduate Program in Rehabilitation Sciences, School of Medicine, University of São Paulo, São Paulo, Brazil; ^2^Hospital Israelita Albert Einstein, São Paulo, Brazil; ^3^Centre for Movement, Occupational and Rehabilitation Sciences, Oxford Brookes University, Oxford, United Kingdom; ^4^Department of Scientific Writing, School of Medicine ABC, Santo André, Brazil; ^5^EACH - School of Arts, Sciences and Humanities, University of São Paulo, São Paulo, Brazil

**Keywords:** Duchenne Muscular Dystrophy, learning, motor skills, virtual reality exposure therapy, virtual reality, computer storage devices, functionality

## Abstract

There is a need to support individuals with Duchenne Muscular Dystrophy (DMD) to achieve optimal functionality in everyday life and with meaningful tasks and activities, throughout stages of the disease progression. Thus, technological developments have created an exciting opportunity for the use of affordable virtual reality (VR) systems with different kinds of interaction devices, providing an efficient and fun tool for enabling improvement in motor performance.

**Objective:** To compare performance on a virtual task using interfaces with and without physical contact in order to identify functionality by using different devices in individuals with DMD.

**Methods:** One hundred and twenty male individuals took part on this study: 60 with DMD with a mean age of 16 ± 5 (range 9–34 years old) and 60 without DMD in the control group (CG) matched by age. Participants were divided into three groups of 20 individuals each which performed a virtual task in three different interfaces: Kinect®, computer Touch Screen and Leap Motion®, in a cross over design in which all participants used all devices. Motor impairment in the DMD group was measured by using the Motor Function Measurement and Vignos scales.

**Results:** All participants improved performance through practice, regardless of the interface used, although the DMD group had a continuous lower performance compared to the CG. In addition, the DMD group obtained a significant better performance with Leap Motion interface compared to the other interfaces, while the CG presented better performance on Touch Screen interface.

**Conclusion:** Leap Motion provided better performance for individuals with DMD due to enablement of distal muscle function and ease of instrument adjustment using the virtual interface. Therefore, this type of interface should be encouraged for promoting functionality on general tasks using computer systems. Clinical Trial register number: NCT02891434.

## Introduction

Duchenne Muscular Dystrophy (DMD) is an inherited recessive genetic disease characterized by the absence of dystrophin protein in muscle fiber membrane resulting from a mutation of the Xp21 gene (1), and has an incidence of ~1 in every 3,500 males (2). DMD is characterized by the progressive and irreversible weakening of muscles that leads to severe physical disability (3). The initial clinical manifestations are muscle weakness of the pelvic and scapular girdles, and muscular contractures and retractions which results in difficulty in walking and other functional activities (4). Individuals with DMD become increasingly dependent on caregivers for daily activities, thus requiring more assistance and care overtime (5, 6). Therefore, there is a need to support individuals with DMD to achieve optimal functionality in everyday life and with meaningful tasks and activities, throughout stages of the disease progression.

Technological developments have created an exciting opportunity for the use of assistive technology for rehabilitation activities through ubiquitous and affordable virtual reality (VR) systems providing a dynamic, enabling, and fun interaction platform (7, 8). For example, computer games were used in the training of respiratory muscles during various stages of disease progression in 15 males with DMD to successfully engage and improve respiratory performance in participants who had moderate impairment in lung function tests (9). Also, a new technique for functional assessment of upper limbs through exercises in virtual environment was evaluated, and results showed that the VR simulator had the ability to assess strength capacity in patients with DMD (10).

A study by Massetti et al. (11) evaluated 22 individuals with DMD to identify whether practicing a task in a virtual environment could improve performance by comparing to a similar task in a real environment, in addition to distinguishing whether there was transference between environments. They concluded that both virtual and real tasks promoted improvement in performance in the acquisition phase, short-term retention, and transfer phase. However, there was no transfer of learning between environments. Although a functional task was not used, the use of virtual environments for individuals with DMD should be considered carefully when the aim is to transfer motor ability from virtual to real environment (11).

In addition to the possibility of using VR in treatment programs to enable improvements in day-to-day tasks, it is important to identify interaction devices that exist in the market that better enable functionality for individuals with DMD to interact with the virtual environment. Individuals with DMD present preservation of the strength of musculature of the upper extremities for longer than in lower extremities, especially distal muscles such as the flexors of the fingers (12). Therefore, adaptations using VR can be used to support functional independence for a longer time period (13) providing various types of interfaces for rehabilitation, and can keep patients engaged with VR tasks. However, no studies have systematically investigated performance using VR nor actual interface devices for individuals with DMD.

Studies illustrated that different interaction devices for the same task could provide a number of significant results (14–16). For instance, using a device such as webcam, Kinect or Leap Motion, provides abstract information (without physical contact) and can result in different performance results compared to the same task performed using a Touch Screen, mouse or computer keyboard that offer more tangible information with physical contact (14, 15). Thus, the aims of the present study were to compare the difference between three interaction devices which may enable improved performance in the execution of virtual tasks in individuals with DMD. Also, to identify if the motor ability acquired with practice on non-contact devices (more virtual, represented by Microsoft's Kinect for Windows and Leap Motion—LMCH, Leap Motion, Inc., San Francisco, CA) would be transferred to contact devices (more real, represented by the Touch Screen) and vice-versa. We also compared differences between DMD and a control group (CG) of healthy individuals after practicing the same task using the different devices.

We hypothesized that non-contact devices would result in worse performance compared to contact devices for both groups as they are typically more difficult to use considering that they have no tactile feedback (11). However, as supposed by Massetti et al. (11), the virtual task (non-contact devices) which is relatively new and more difficult for the participants, will enable an experience that provides new adaptations and motor engrams during acquisition, and is considered beneficial for providing better transfer of performance to a real environment situation (or an environment with more real life characteristics such as a Touch Screen that provides tactile feedback); with the CG predicted to have better performance for all interfaces (17, 18). If this hypothesis is correct, results will provide practical benefits in helping health professionals to use the best devices on VR to enable individuals with DMD to be more functional and independent as the disease progresses.

## Methods

This was a randomized repeated exposure controlled trial and was approved by the Ethics Committee for review of research projects of the School of Medicine, University of São Paulo (FMUSP), under protocol number 248/15 and registered at Clinical Trials under register number NCT02891434. The participants and/or the legal guardians of the children under the age of 18 years old provided written informed consent.

### Participants

A total of 120 individuals took part in this study, 60 with DMD and were recruited through the Brazilian Association of Muscular Dystrophy (ABDIM)—(age 16 ± 5 years old, ranging between 9 and 34 years old) and 60 healthy males for the Control Group (CG), matched by age (age 16 ± 5 years old, ranging between 9 and 34 years old).

The inclusion criteria for the DMD group were a confirmed diagnosis of DMD. Those in the CG were males without any neuromuscular conditions. Exclusion criteria for participants were: not capable of performing the virtual task after one test-trial, in which we assessed the comprehension of the task by assessing performance in this single trial (verbal and written instructions were provided before the experiment), or presence of upper limb deformity or muscle weakness that prevented handling of the devices used in this study. However, no participant was excluded based on these criteria.

To describe and characterize motor impairments in the DMD group, the Motor Function Measure (MFM) scale was used (19). The scale is subdivided into three dimensions: dimension 1 (D1) standing position and transfers (13 items); dimension 2 (D2) axial and proximal limb motor function (12 items); dimension 3 (D3) distal limb motor function (7 items). Items are scored from zero to three with a total percentage value obtained by adding all section percentage totals (20). To describe the staging of the disease the Vignos scale was administered with a score of 1–10, and a higher value equating to more severe disease (21, 22).

### Instruments

After providing instructions for the interventions, participants performed the task individually in a quiet room with only the experimenter present. The computer and monitor were placed on a flat table. The participants were seated on a chair (ambulant) or seated in their own wheelchair, which was adjusted to height according to the needs of the individual. The experimenter explained and demonstrated the task and participants were instructed to place the preferred hand (i.e., the less affected hand) with all three interfaces in a random order.

To evaluate the performance of the interaction devices, we used a computer game proposed by the Department of Information Systems, University of São Paulo (23). The game was chosen due to its low cognitive demands and ease and adaptability for use in individuals with DMD.

Three interfaces were investigated, two without physical contact and one with physical contact. The first of these was represented by Kinect for Windows (Microsoft, Redmond WA, USA), which consists of a sensor that captures body movements (including upper limbs) (24). The second non-physical contact interface was Leap Motion (LMCH, Leap Motion, Inc., San Francisco, CA, USA), a virtual interface by means of a sensor and its catchment area is focused only on hands and fingers (29). The third interface, requiring physical contact, was a Touch Screen monitor on the computer screen itself (23).

**Figure 1:**
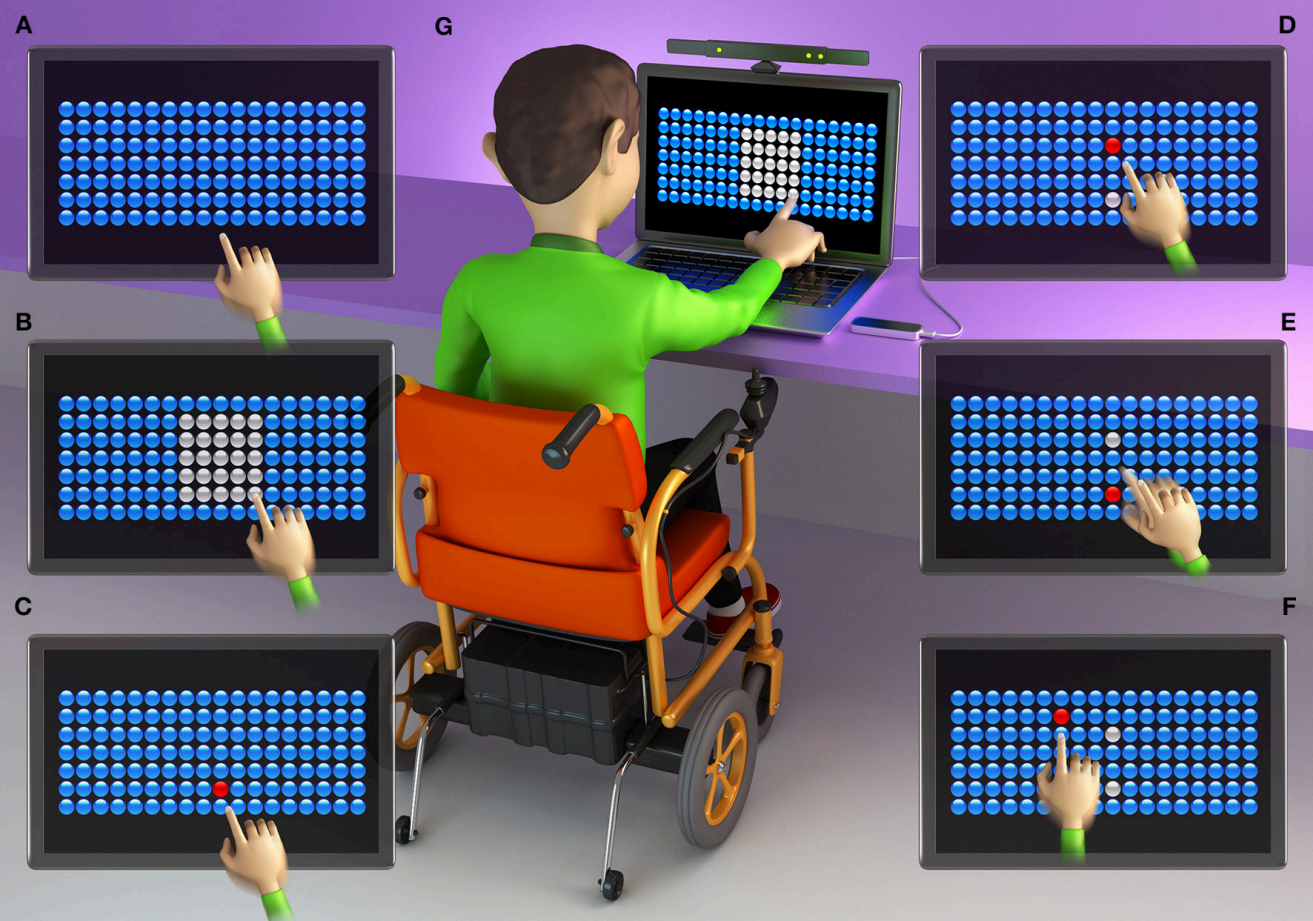
Graphic representation of an individual with DMD using the Touch Screen interface. **(A)** initial screen of the task, with 126 bubbles; **(B)** participant defines the area of the range zone by touching the screen for 10 s; **(C)** participant touches the first target bubble (defined by the researcher in the center of the bottom line of the range zone); **(D)** participant touches a bubble that appears at random (within the range zone); **(E)** participant returns to touch the target bubble; **(F)** some touches of the bubble are outside of the range zone, challenging the limits of the participant; **(G)** individual with Duchenne Muscular Dystrophy (DMD) during the task using the touch screen interface.

**Figure 2:**
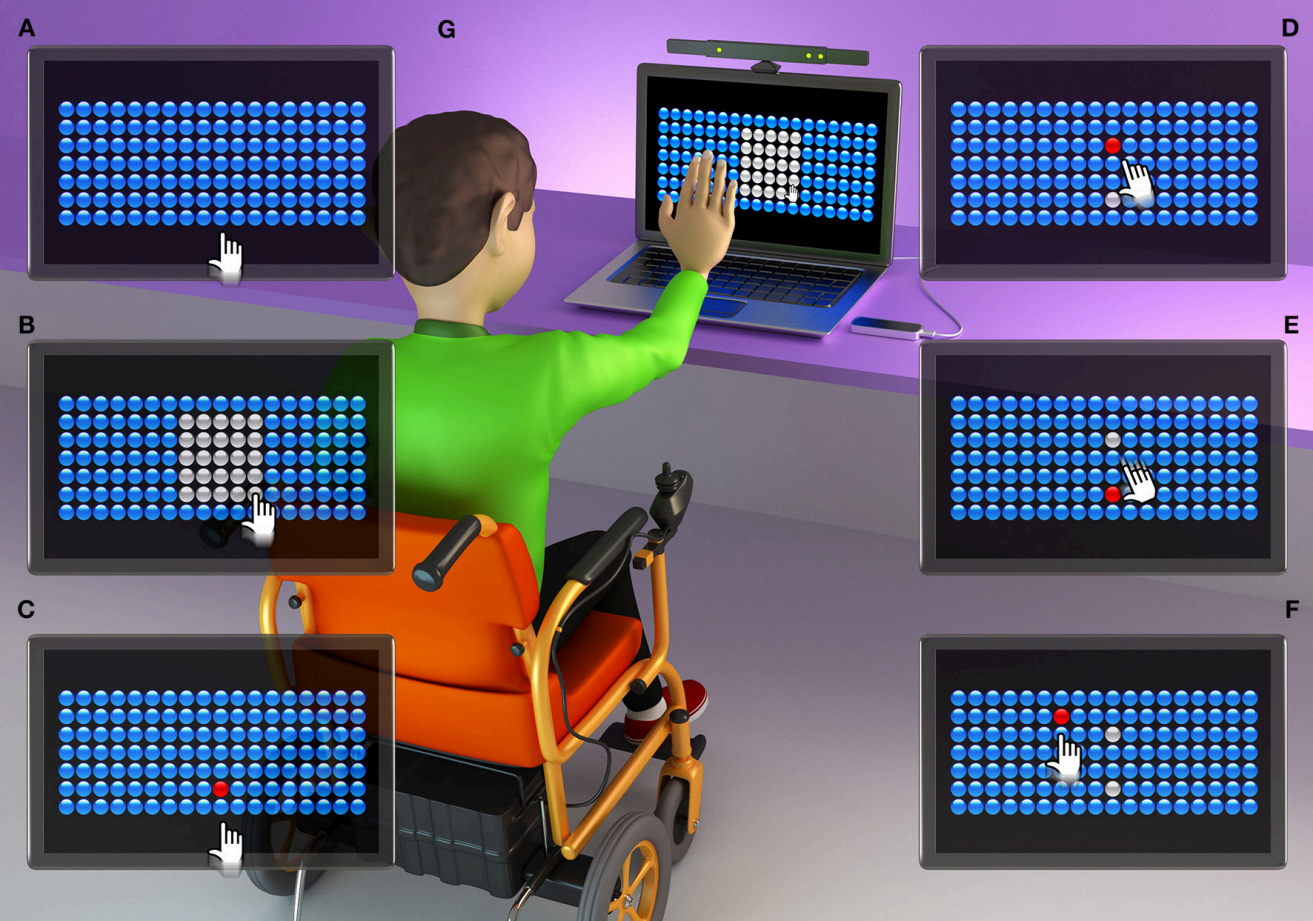
Graphic representation of an individual with DMD using the Kinect interface. **(A)** initial screen of the task, with 126 bubbles; **(B)** participant defines the area of the range zone for 10 s; **(C)** participant touches the first target bubble (defined by the researcher in the center of the bottom line of the range zone); **(D)** participant touches a bubble that appears at random (within the range zone); **(E)** participant returns to touch the target bubble; **(F)** some touches of the bubble are outside of the range zone, challenging the limits of the participant; **(G)** individual with Duchenne Muscular Dystrophy (DMD) during the task, using the Kinect interface. Some participants needed to support the upper limb on the arm of the wheelchair.

**Figure 3:**
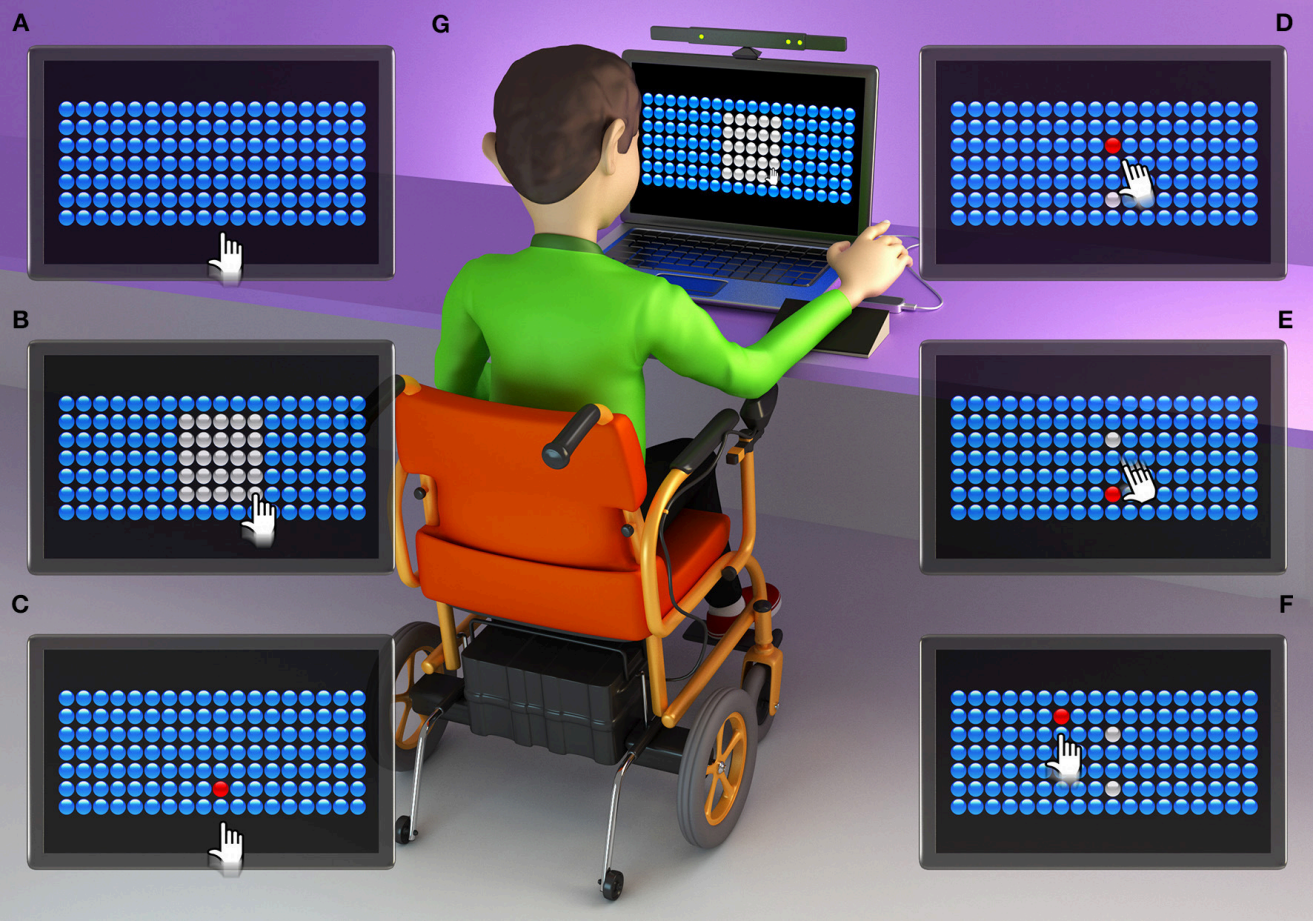
Graphic representation of an individual with DMD using the Leap Motion interface. **(A)** initial screen of the task, with 126 bubbles; **(B)** participant defines the area of the range zone for 10 s; **(C)** participant touches the first target bubble (defined by the researcher in the center of the bottom line of the range zone); **(D)** participant touches a bubble that appears at random (within the range zone); **(E)** participant returns to the bubble target; **(F)** some touches of a bubble are outside of the range zone, challenging the limits of the participant; **(G)** individual with Duchenne Muscular Dystrophy (DMD) during the task, using the Leap Motion interface (when necessary a wedge was used to adapt the lifting of the handle).

The computer game presents 126 bubbles arranged in rows and columns (Figures 1A, 2A, 3A). Participants must attempt to change the color of the largest number of bubbles in 10 s, thereby defining a range zone (Figures 1B, 2B, 3B).

After defining the range zone, the therapist established a red target bubble, which was chosen in the center of the bottom line of the range zone (Figures 1C, 2C, 3C). After touching the target bubble, the game had another red bubble in a random position, within the area of the range zone (Figures 1D, 2D, 3D). After reaching the second bubble, the bubble target was displayed again in another location, and so on (Figures 1E, 2E, 3E). The game features red bubbles within the area of the range zone (Figures 1E, 2E, 3E), and sometimes out of this zone (Figures 1F, 2F, 3F), thus creating a higher degree of difficulty and encouraging participants to challenge their limits (23), using touch screen (Figure 1G), Kinect (Figure 2G) or LeapMotion interface (Figure 3G).

### Procedure and Design

During performance using the Leap Motion sensor, a wedge to support the handle was used leaving a required distance for capturing the movement of the fingers. For the Kinect interface, some participants needed support for the upper limb on the arm of the wheelchair. This was done due to individuals with DMD needing to sustain the upper limb for an extended time, which can lead to muscle fatigue and consequently can decrease the performance on the task.

**Figure 4:**
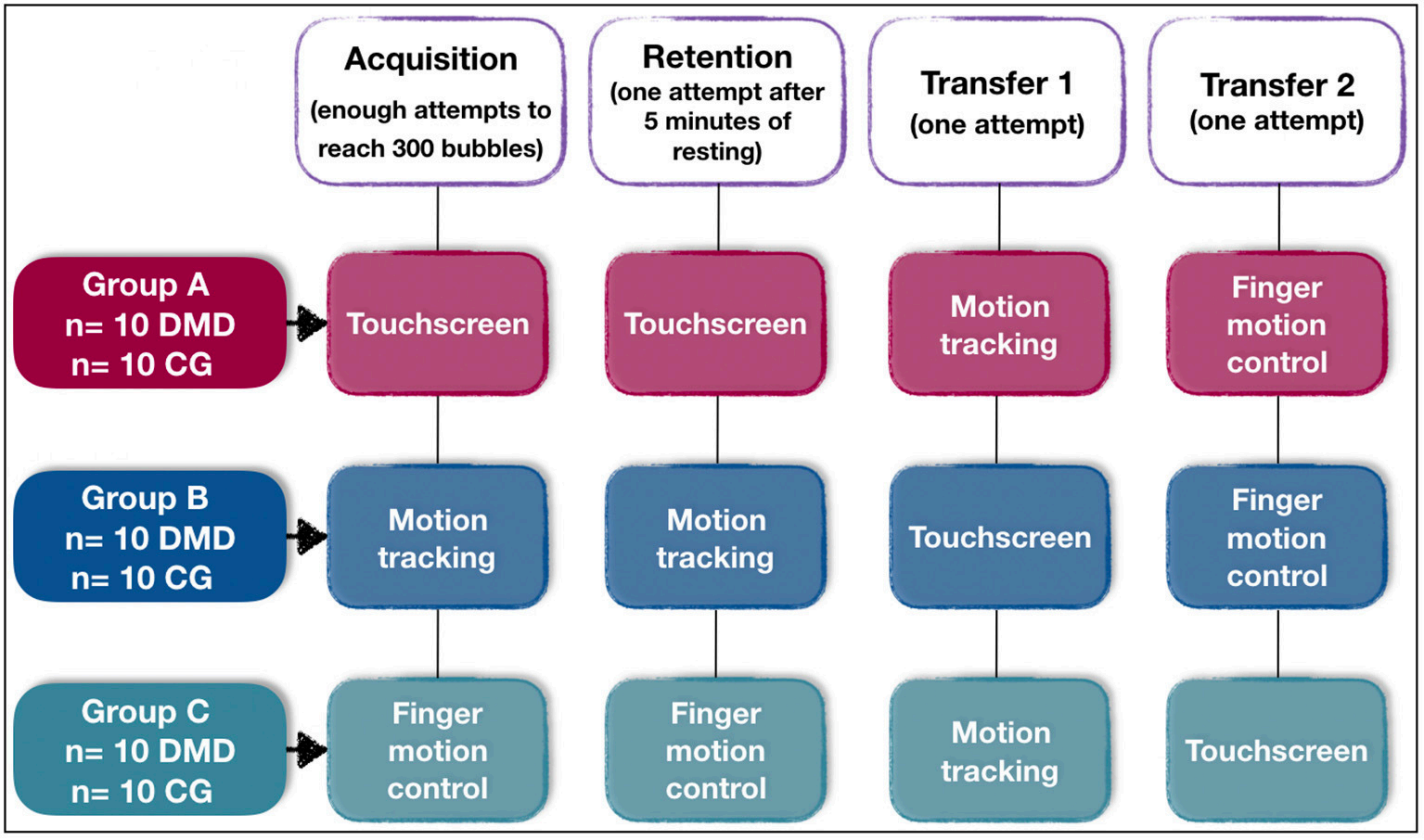
Outline of the experimental and control groups during the phases of acquisition, retention and transfer, and their interfaces. Abbreviations: *n*, number of participants; DMD, Duchenne muscular Dystrophy; CG, control group.

In order to assess the performance of individuals from both groups for each device, a short-term motor learning design was carried out. This considered three phases: acquisition in which the participants practiced the task with enough attempts to improve performance, in addition to assessing the capacity to retain the improvement acquired after a period with no contact with the task (retention test). It was also determined if the participant would maintain their performance when changing some characteristics of the task (transfer test) (25). Thus, all participants performed the same task in four stages in a cross-sectional design: acquisition (enough attempts to reach 150 bubbles), retention (one attempt after 5 min with no contact with the task) and transfer (transfer 1 and 2—one attempt in each device that was not used in the acquisition, see Figure 4). The entire trial took approximately 30 min. In this randomized cross over trial the participants (DMD and CG) were randomly allocated to 1 of 3 groups: Group A, which underwent acquisition and retention using the Touch Screen interface with transfers onto the Leap Motion and Kinect interfaces, respectively; Group B, which underwent acquisition and retention using the Kinect interface with transfers on the Touch Screen and Leap Motion interfaces, respectively; Group C: which underwent acquisition and retention using the Leap Motion interface with transfers on the Touch Screen and Kinect, respectively (Figure 4).

All participants performed the task with all interfaces.

### Data Analysis

A one-way ANOVA was performed on the difference in the mean scores for the functional scales (MFM-D1, MFM-D2, MFM-D3, MFM-tot, Vignos) and age for the DMD group, to attest the homogeneity of the sample within groups of interfaces (Table 1).

**Table 1:** Mean and standard deviations of the groups in each functional scale scores category.

		**Age**	**Vignos**	**MFM-D1**	**MFM-D2**	**MFM-D3**	**MFM-Tot**
**Gr**	***n***	**M (SD)**	**M (SD)**	**M (SD)**	**M (SD)**	**M (SD)**	**M (SD)**
K	20	15.9 (5.6)	6.1 (2.2)	17.7 (26.7)	59.3 (29.8)	84.1 (10.6)	54.6 (18.4)
LM	20	17.0 (4.7)	6.2 (2.5)	15.4 (23.7)	59.7 (24.3)	78.3 (17.3)	61.0 (23.7)
TS	20	17.4 (4.6)	6.3 (2.5)	13.1 (22.4)	60.1 (27.1)	72.5 (24.1)	47.6 (21.7)
*p*		0.64	0.97	0.86	0.99	0.21	0.18
Total		16.65 (4.90)	6.23 (2.32)	15.23 (23.9)	61.05 (30.7)	78.06 (18.8)	54.38 (21.9)
Min/Max		9/34	1/8	0/74	0/100	19.5/100	13.54/87.5

*In addition, the p-value of one-way ANOVA within groups of interfaces. Gr, group; K, Kinect; LM, Leap Motion; TS, Touch Screen; n, number of participants; M, mean; SD, standard deviation; MFM, Motor Function Measure; MFM-D1, first domain score of the MFM scale; MFM-D2, second domain score of the MFM scale; MFM-D3, third domain score of the MFM scale; MFM-Tot, total domain score of the MFM scale; Min, minimum; Max, Maximum*.

The number of bubbles reached was considered the dependent variable and was submitted to a 2 (group: DMD, CG) by 3 (Interfaces: Kinect, Touch Screen, Leap Motion) by 2 (Attempt) ANOVA, with repeated measures on the last factor. For the factor *Attempt*, separate comparisons were made for acquisition (first acquisition attempt—FA vs. last acquisition attempt—LA), retention-R (LA vs. retention attempt—R) and transfer-T (R vs. transfer attempt T1, R vs. transfer attempt T2). *Post-hoc* comparisons were carried out using Tukey-LSD (Least Significance Difference). Partial eta-squared (η_p_^2^) was reported to measure effect size and was interpreted as small (effect size >0.01), medium (effect size >0.06), or large (effect size >0.14) (26).

Regression analysis was performed to determine if factors including age, MFM-D1, MFM-D2, MFM-D3, MFM-total, and Vignos influenced performance during the practice test for the DMD group, considering improvement in number of bubbles touched (BT) from the first to final practice blocks (difference LA-FA). The software used was SPSS, 20.0. Significance was set at *p* < 0.05.

## Results

Demographics of the DMD group including age and functional scale score are described in Table 1, showing the homogeneity within groups of interface. Table 2 shows the number of individuals in each item on the Vignos scale.

**Table 2:** Classification of the level of severity of Duchenne Muscular Dystrophy (DMD) using the Vignos scale, within groups of Interfaces and total.

	**Vignos scale**	**Number of individuals**			
		**K**	**LM**	**TS**	**Total**
1	Walks and climbs stairs without assistance	1	1	1	3
2	Walks and climbs stairs with aid of railing	1	2	2	5
3	Walks and climbs stairs slowly with aid of railing (over 25 s for 8 standard steps)	1	1	1	3
4	Walks unassisted and rises from chair but cannot climb stairs	0	0	0	0
5	Walks unassisted but cannot rise from chair or climb stairs	0	1	0	1
6	Walks only with assistance or walks independently with long leg braces	2	0	0	2
7	Walks in long leg brace but requires assistance for balance	8	5	6	19
8	Stands in long leg brace but enable to walk even with assistance	3	8	8	21
9	Is in a wheelchair	0	0	0	0
10	Is confined to a bed	0	0	0	0

*n = 54, because six of them had not Vignos scores*.

### Acquisition

For acquisition we compared first acquisition attempt—FA with the last acquisition attempt—LA, in order to discover improvements by considering the amount of bubbles reached during practice.

**Figure 5:**
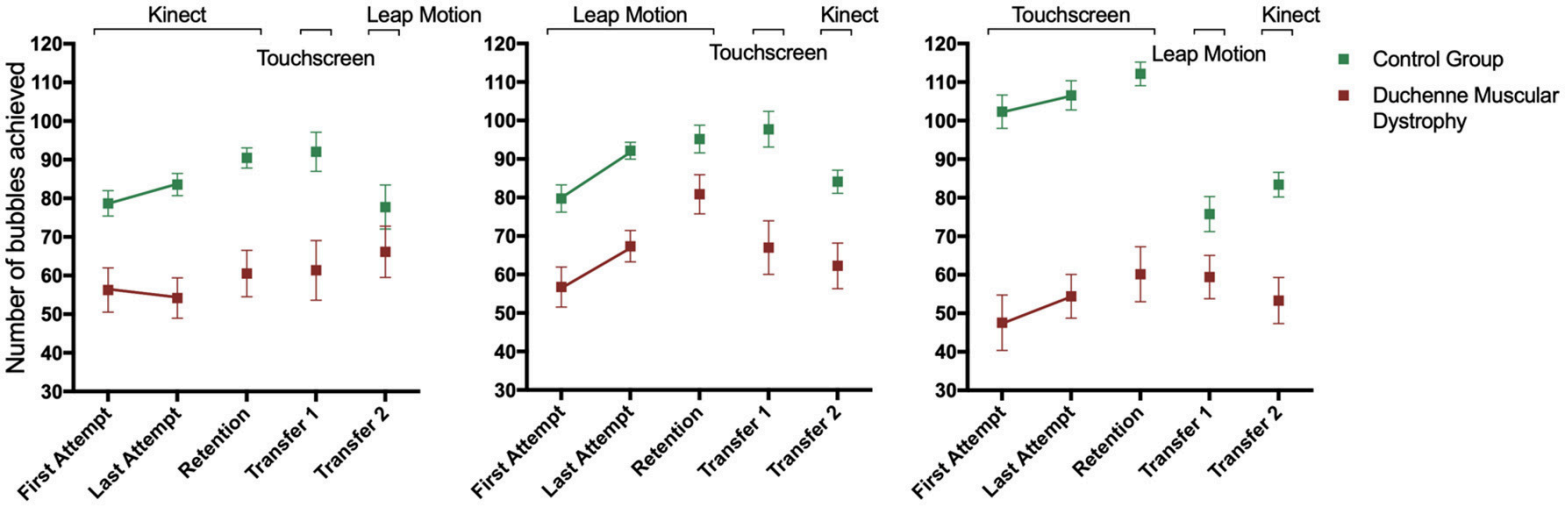
Number of bubbles touched by Duchenne Muscular Dystrophy (DMD) and the control group (CG) during the stages of motor learning.

Significant effects were found for Attempt, [*F*_(1, 114)_ = 20.8, *p* < 0.001, η_*p*_^2^ = 0.15, Group, *F*_(1, 114)_ = 92.6, *p* < 0.001, η_*p*_^2^ = 0.45, but not for Interface *F*_(2, 114)_ = 2.3, *p* > 0.05, $\eta_{\text{p}}^{2}$ = 0.04]. Therefore, both groups increased the number of BT from First Attempt (FA) (*M* = 70) to Last Attempt (LA) (*M* = 78). The DMD group had worse performance (*M* = 57) when compared to CG (*M* = 91). Interaction for group by interface [*F*_(2, 114)_ = 7.4, *p* = 0.001, η_*p*_^2^ = 0.12] was found indicating a difference between the DMD and CG with DMD performing worse on all interfaces, on Touch Screen (*M* = 105 and 52, respectively), Leap Motion (M = 86 and 62, respectively), and Kinect (*M* = 81 and 57, respectively). The results are represented in Figure 5.

In the CG, the performance was better for Touch Screen (*M* = 105) when compared to Leap Motion (*M* = 86) and Kinect (*M* = 81), with no difference between Kinect and Leap Motion. In DMD group, the performance was worse on Touch Screen (*M* = 50) when compared to Kinect (*M* = 54) and Leap Motion (*M* = 62). In addition, the *post-hoc* test also showed that the DMD group had a better performance in the LA block on Leap Motion (*M* = 67) compared to the Kinect (*M* = 54) and Touch Screen (*M* = 54). For the CG there was no difference between interfaces in the different blocks.

### Retention

The last acquisition attempt was compared—LA vs. Retention—R, to assess the short-term retention of performance acquired with practice, after a 5 min break with no contact with the task.

Significant effects were found for Attempt, [*F*_(1, 114)_ = 15.4, *p* < 0.001, η_*p*_^2^ = 0.12, Group, *F*_(1, 114)_ = 90.1, *p* < 0.001, η_*p*_^2^ = 0.44, and Interface *F*_(2, 114)_ = 4.2, *p* = 0.0017, η_*p*_^2^ = 0.07]. Similarly to the acquisition phase, these results suggest that the participants increased the number of BT from Last Attempt (LA) (*M* = 78) to Retention (R) (*M* = 83), with CG showing better performance (*M* = 97) when compared to the DMD group (*M* = 62). In addition, interaction for group by interface [*F*_(2, 114)_ = 7.3, *p* = 0.001, η_*p*_^2^ = 0.11] was found meaning that the CG presented better performance compared to the DMD group in all interfaces: on Touch Screen (*M* = 109 and 56, respectively), on Leap Motion (*M* = 94 and 74, respectively) and on Kinect (*M* = 87 and 56, respectively). *Post hoc* analysis found a significant difference for the DMD group from LA to R on Leap Motion (*M* = 67 and 81, respectively).

### Transfer 1

For transfer 1 and 2, comparisons were made between Retention-R vs. Transfer 1, and R vs. Transfer 2, to assess the transfer of performance to different types of interaction devices.

Significant effects were found for Attempt, [*F*_(1, 114)_ = 11.8, *p* = 0.001, η_*p*_^2^ = 0.09 and Group, *F*_(1, 114)_ = 67.0, *p* < 0.001, η_*p*_^2^ = 0.37]. These results suggest that the participants decreased the number of BT from R (*M* = 83.2) to T1 (*M* = 74.7), and the CG had a larger number of BT (*M* = 94) compared to the DMD group (*M* = 64). Interaction for attempts by interface, [*F*_(2, 114)_ = 5.1, *p* = 0.007, η_*p*_^2^ = 0.01], was found. *Post-hoc* analysis showed that both groups (DMD and CG) that performed the acquisition and retention phases on Touch Screen (Retention *M* = 86) decreased performance on transfer for Leap Motion (*M* = 68). There was no significant difference between acquisition/retention on Leap Motion (*M* = 88.2) and transfer for Touch Screen (*M* = 80), nor for acquisition/retention on Kinect (M = 75) and transfer for Touch Screen (*M* = 76). Considering the performance for each group, in the CG group alone there was difference in acquisition/retention on Touch Screen (*M* = 112) and with worse performance on transfer on Leap Motion (*M* = 76), and in the DMD group there was difference in acquisition/retention performance on Leap Motion (*M* = 81) with worse performance for transfer on Touch Screen (*M* = 59).

### Transfer 2

Significant effects were found for Attempt, [*F*_(1, 113)_ = 30.7, *p* < 0.001, η_*p*_^2^ = 0.21, and Group, *F*_(1, 113)_ = 55.2, *p* < 0.001, η_*p*_^2^ = 0.33]. These results suggest that the participants decreased the number of BT from R (*M* = 83) to T2 (*M* = 71). The CG group had a larger number of BT (*M* = 90) than the DMD group (*M* = 64). Interaction for attempts by group, [*F*_(1, 113)_ = 6.1, *p* = 0.015, η_*p*_^2^ = 0.05], attempts by interface, [*F*_(2, 113)_ = 4.5, *p* = 0.013, η_*p*_^2^ = 0.07], and group by interface, [*F*_(2, 113)_ = 4.2, *p* = 0.017, η_*p*_^2^ = 0.07, were found. *Post-hoc* analysis showed that both groups that performed the acquisition and retention phases on Touch Screen (Retention *M* = 86) decreased performance on transfer for Kinect (*M* = 68). Additionally, there was significant difference between acquisition/retention on Leap Motion (Retention *M* = 88) and transfer for Kinect (*M* = 73), but there was no significant difference between acquisition/retention on Kinect (Retention *M* = 76) and transfer for Leap Motion (*M* = 73).

### Regression Analysis

The regression analysis revealed that the scores of the Vignos (positively related) and MFM-D2 (inversely related) scales predicted the improvement of performance in the DMD group, while age, MFM-D1, MFM-D3, and MFM total did not [*F*_3, 26_ = 3.65, *p* = 0.025, *r*_2_ = 0.30], equation: Improvement = 26.1 ^*^Vignos, −0.857 ^*^MFM-D2].

## Discussion

This study found that people with DMD benefitted from the use of virtual technologies as compared to touch interfaces in acquisition and retention of performance during tasks. Contrary to what we hypothesized that non-contact devices would result in worse performance compared to contact device, the results demonstrate that in individuals with difficulty in moving the upper limbs, there is an improvement in performance when using the Leap Motion interface that enabled distal muscle functioning and ease of instrument adjustment for individuals with DMD. This result is supported by Massetti et al. (11) who demonstrated that individuals with DMD are able to learn new tasks in real and virtual environments and are able to retain and transfer the performance. Our findings suggest that interfaces with no physical contact may offer an exciting opportunity to support independent functioning across a number of meaningful activities in this group. As independent functioning, we can speculate that Leap Motion or similar devices can be used with computers to allow interaction and socializing with others, and also to do routine tasks such as internet banking, study, watching TV and in future, virtual movements can be used to control a wheelchair. There is a need to further explore the use of VR for people with DMD and other disorders in which movement is affected, in order to determine strategies for optimal functioning for individuals and throughout stages of disease progression.

During the acquisition phase, regardless of the interface used, there was improvement in task performance with practice, comparing the first attempt (FA) to the Last Attempt (LA) for both groups, and also in the retention phase—in which the participants increased the number of the bubbles reached from the Last Attempt (LA) to the Retention (R). We observed that practice improved performance possibly due to improvements in quality and organization of movement (14).

Despite the improved performance in the acquisition and retention phase in both groups, the DMD group had worse performance in the acquisition phase and in the retention phase when compared to CG. Malheiros et al. (18) used a computer maze task and found similar results, demonstrating that despite its ability to improve performance in a motor task on the computer, individuals with DMD had impaired functional performance compared to CG. Nakafuji and Tsuji (27) and Cyrulnik et al. (28), observed improvement in the learning curve, with the DMD group consistently having slightly worse performance than the CG. The difficulties of individuals with DMD compared to the CG could be due to the deficit, both in adaptive functioning measures, such as cognition, receptive and expressive language, visual-spatial skills, fine motor skills, attention and memory (28).

Regarding performance on each device in the acquisition and retention phases, the best performance in the DMD group was using the Leap Motion interface. Considering the typical preservation of wrist and finger functional capacity which lasts for a longer time period in DMD compared with other limb functions (12), the Leap Motion technology offered better utilization of these distal movements and thereby justifies its use as an interface for enhancing functionality in DMD. The findings support that alternative technologies may offer more optimal solutions compared to standard rehabilitation techniques. The Leap Motion capture by fine movements of the hand joints may be a suitable tool for providing thin manual functions (29, 30).

As hypothesized, we found that performance in the CG was best when using the touch screen interface and therefore they benefited from the possibility of haptic information and response speed offered by the Touch Screen. A task that involves a direct interaction with the environment, including physical contact (more real task) provides a richer set of information to guide the movement compared to a more abstract task without physical contact (more virtual task) (15).

Individuals with DMD have weakness in segments such as shoulders and elbows, which hinder the movement of pronation and supination of the forearm, in addition to the radial and ulnar deviation (31–33). We can only speculate that these conditions hinder performance at the interface with touch screen, which requires greater power and control of the proximal trunk muscles (30).

Finally, considering the transfer phase, all participants had worse performance regardless of the interface used. When the CG started the task using the interface with physical contact and transferred to non-contact (Leap Motion), there was deterioration in performance. Again, the difficulty in accomplishing the task without the benefits of physical contact is probably responsible for the lower performance in the Leap Motion interface. However, in the DMD group the results were opposite, and there was a decrease in performance when transferring to a more real environment feature (Touch Screen). This was previously observed by Monteiro et al. (15) who found that practice in virtual interfaces resulted in a decline in performance when transferring to environments with Touch Screen interfaces.

Another important finding was that age did not influence the learning effects for the gaming task. Such results corroborate with the findings of Malheiros et al. (18), in which age also did not influence performance in a computer task in the DMD group. This is an unexpected result as the disease progress and motor function deteriorate with age.

Furthermore, while exploring the relationship between motor function and performance, no relationship was found between the functionality of the hands (Dimension D3 of MFM) and performance. Mattar and Sobreira (34) indicated that the strength of the hands in individuals with DMD decreases with age and is significantly different compared to the typically developing adults. However, as our sample was composed mostly of adolescents who had good scores on the MFM-D3 (around 80%—Table 1), we can speculate that there was not enough impairment in distal function that could influence the performance on the task. Possibly the VR interface acted to equalize deficits and thus shows the promise for VR interfaces such as the Leap Motion to enable functioning in conditions such as DMD. In addition, we also found that trunk motor skills (Dimension D1 of MFM) did not influence performance in any of the tasks. Controversy, the study by Capelini et al. (17) showed that trunk functionality influenced performance on a virtual task using a Smartphone i.e., individuals with lower scores on functionality performed worse on the task. However, individuals in the study by Capelini et al. (17) presented a lower mean score of MFM-D1 (~12%), compared to the current population of DMD (~15%).

Nonetheless, we do report a relationship between a greater difference between LA and FA (higher learning curve) and the higher score on the Vignos scale and lower score on Dimension D2 of MFM. This means that individuals with more severe motor impairment and lower proximal musculature motor skills had greater capacity to adapt to the task and tended to have greater learning, which may be due to the fact they started with lower scores.

A limitation of the study was that the data was not extrapolated to function in daily life activities, and we used only one specific game to verify the performance on three different devices. Another limitation is the fact that we have not evaluated usability or compliance of individuals, in order to clarify engagement and the influence of their judgement in the performance of the task. As we have found positive results regarding Leap Motion, we suggest that future studies should use different tasks and/or games to assess performance, as well as use scales to assess usability and compliance to provide explanations regarding function in every day use.

## Conclusion

The results showed an improvement in performance when using a virtual interface requiring no physical contact for individuals with DMD, despite the progressive difficulty in movement and strength of the upper limbs. A key factor in the rehabilitation and care of individuals with DMD is to identify ways to facilitate performance in activities of daily living and to encourage the development and maintenance of motor skills with emphasis on upper limbs (4). Thus, a device with no contact (Leap Motion) facilitated the successful implementation of the proposed task, which provides evidence for future research using virtual interfaces which encourage distal movement and have the potential allow for improvements in daily tasks for individuals with DMD.

## Author Contributions

BdF structured the manuscript, directed the work, performed the data collection, and organized the data. TdS structured the method and results, performed data analysis, and revised the manuscript. TC performed data analysis. TM structured the manuscript. LdA developed the software and structured the database. SC and HD revised the manuscript and adapted the work to the English language. FC revised the manuscript. CM mentored the work, organized, and revised the manuscript.

### Conflict of Interest Statement

The authors declare that the research was conducted in the absence of any commercial or financial relationships that could be construed as a potential conflict of interest.
